# E1A-engineered human umbilical cord mesenchymal stem cells as carriers and amplifiers for adenovirus suppress hepatocarcinoma in mice

**DOI:** 10.18632/oncotarget.10122

**Published:** 2016-06-17

**Authors:** Zhenzhen Li, Zhou Ye, Xiaolong Zhang, Qing Zhang, Dongmei Fan, Yanjun Zhang, Hongbo R. Luo, Xiangfei Yuan, Zongfang Li, Dongsheng Xiong

**Affiliations:** ^1^ State Key Laboratory of Experimental Hematology, Institute of Hematology & Hospital of Blood Diseases, Chinese Academy of Medical Sciences & Peking Union Medical College, Tianjin 300020, China; ^2^ National-Local Joint Engineering Research Center of Biodiagnostics & Biotherapy, The Second Affiliated Hospital, Xi'an Jiaotong University, Xi'an 710004, China; ^3^ Central Hospital of Karamay, Karamay, Xinjiang 834000, China; ^4^ Department of Pathology, Joint Program in Transfusion Medicine, Harvard Medical School, and Department of Laboratory Medicine, Children's Hospital Boston, Boston, MA 02115, USA; ^5^ Tianjin Institute of Integrative Medicine for Acute Abdominal Diseases, Nankai Hospital, Tianjin 300100, China

**Keywords:** HUMSC, adenovirus delivery, gene therapy, hepatocellular carcinoma

## Abstract

Gene therapy is an attractive approach for hepatocellular carcinoma (HCC) patients. Nevertheless, efficient transgene delivery remains a challenge. In this study, we explored a new targeted system based on human umbilical cord-derived mesenchymal stem cells (HUMSCs), which were engineered to deliver adenovirus to tumor sites, and to replicate and assemble into new adenovirus against HCC. Our results showed that HUMSCs infected by Ad-hTERTp-IL24 followed by LentiR.E1A infection could specifically migrate to HepG2 tumor cells and support adenoviral replication in vitro and in vivo 36 h after LentiR.E1A infection. Ad-hTERTp-IL24 specifically inhibited HepG2 cells growth, and this inhibitory effect was enhanced by low doses of 5-fluorouracil (5-Fu), because the expression levels of coxsackie adenovirus receptor (CAR) and integrin ανβ3 on tumor cells were significantly increased, causing higher viral uptake. Compared with the no treatment groups, Ad-hTERTp-IL24 and LentiR.E1A co-loaded HUMSCs exhibited significant anti-tumor activity in vivo, particularly in combination with low doses of 5-Fu. In summary, this study provides a promising targeted gene therapeutic strategy dependent on the tumor tropism of HUMSCs, to improve the outcome of virotherapy for tumor patients especially those with metastatic diseases.

## INTRODUCTION

Gene therapy is an attractive and promising approach to cancer treatment. Currently, adenoviral vectors have been employed for gene therapy due to their low pathogenicity, high titer and lack of integration into the host cells' genomes. The “first generation” vectors, replication-deficient vectors based on human adenovirus serotypes 5 (Ad5), in which the E1 and E3 regions of the genome are deleted, are the most widely used [1]. Clinical studies have shown that administration of replication-deficient adenoviral vectors intratumorally, intraperitoneally and intravesically is a safe, feasible and effective antitumor strategy against many types of cancers [2]. Nevertheless, the major concerns over the use of such vectors lie in the inefficient viral delivery to the metastatic tumor sites due to the fact that the metastatic tumors are often smaller and directly inaccessible. Moreover, systemic administration of high doses of adenovirus is associated with systemic toxicity and rapid elimination of the virus by the immune system before reaching the tumor [3]. Thus, it is absolutely critical to develop an efficient and targeted delivery system for intravenous administration of adenoviral vectors to improve the clinical outcome of patients with recurrent and metastatic lesions.

Mesenchymal stem cells (MSCs) have been shown to migrate toward malignant tumors and track microscopic metastasis when administered by intravenous injection in vivo [4, 5]. Further, engineered MSCs have been indicated as a potential vehicle to deliver anticancer agents to primary and metastatic tumors [6–8]. At present, scientists have successfully taken advantage of MSCs to deliver antitumor agents, including cytokines [5], interferons [9], pro-drugs [10] and conditionally replicating virus [11]. Human umbilical cord's Wharton's jelly (WJ)-derived mesenchymal stem cells (HUMSCs), were first described by McElreavey *et al* [12]. HUMSCs share common characteristics of MSCs, such as immunosuppression, expression of a phenotypically defined set of surface markers (CD90, CD105 and CD73), multi-differentiation potential to the osteogenic, adipogenic and chondrogenic lineages [13], and ability to accumulate at sites of tissue damage, inflammation and tumors in vivo [6]. In addition, HUMSCs are advantageous in term of rapid cell expansion, yield, ease of procedure, lack of ethical problems and are suitability for genetic engineering with viral vectors [14]. These characteristics make HUMSCs to be a promising platform for targeted delivery of anticancer agents for a variety of cancers.

To enhance the transfer of adenovirus to tumor cells, we engineered the HUMSCs to produce an adenovirus encoding antitumor agents. The replication-deficient adenoviral vectors based on Ad-5 can be propagated in complementing human cell lines that provide the E1 proteins [15]. Therefore, if E1A proteins, which are essential for the replication of the adenovirus, were supplied in HUMSCs, the HUMSCs would permit replication-deficient adenoviruses to be replicated and packaged [16]. To this end, HUMSCs were first infected by replication-deficient adenoviruses harboring the antitumor gene, and then modified by lentiviruses expressing E1A proteins. The engineered HUMSCs not only delivered adenoviral vehicles to tumor or metastatic tumor sites but also supported the adenoviral replication. Ultimately, the recombinant adenovirus was amplified and packaged, and released, allowing the infection of the surrounding tumor cells to express the target therapeutic proteins.

In this study, melanoma differentiation associated gene-7/interleukin-24 (mda-7/IL-24) was chosen as the target therapeutic protein carried by the adenoviral vectors. IL-24, a member of the IL-10 family, can selectively induce apoptosis in a variety of cancer cells without affecting the normal cells in vitro [17, 18], in vivo [17, 19, 20], and in a phase I clinical trial [21, 22]. Moreover, untransfected neighboring cancer cells can be killed by the bystander effect of mda-7/IL-24 [23, 24]. To enhance the secondary anti-tumor specificity, the expression of IL-24 is driven by the human telomerase reverse transcriptase (hTERT) promoter, which is highly active in over 85% of human cancer cells but inactive in most somatic cells. The hTERT promoter has shown great potential in regulating the cell-specific expression of exogenous therapeutic genes in tumor cells without influencing the normal tissues [25, 26].

We evaluated the efficacy of this dual targeting therapeutic system in vitro and in vivo in hepatocarcinoma models. Our results showed that adenovirus-loaded HUMSC.lentiR.E1A could support adenoviral replication and viral particle release to infect the tumor cells. Moreover, virus-loaded HUMSCs were still capable of migrating to hepatocellular carcinoma. The tumor suppressive effect of this dual targeted therapeutic system was also observed in vitro and in vivo. Furthermore, we investigated the synergistic antitumor effect of this dual targeted therapeutic system in combination with 5-fluorouracil (5-Fu).

## RESULTS

### Culture and identification of HUMSCs

**Figure 1:**
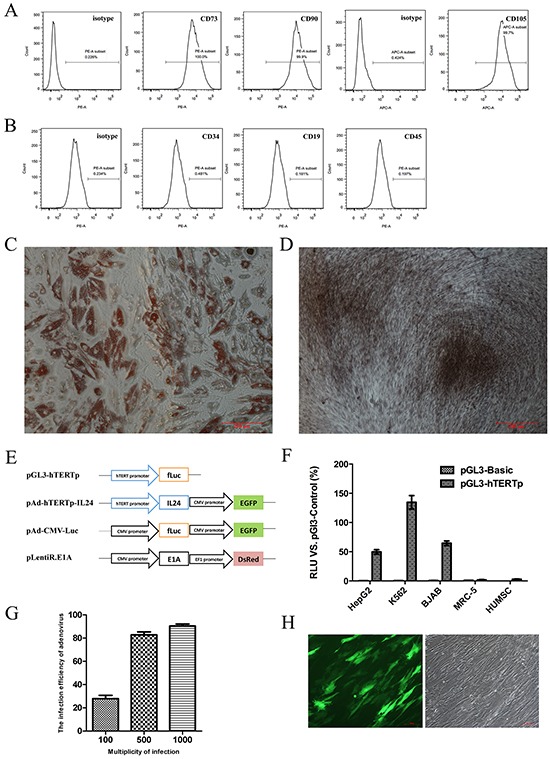
Identification of HUMSCs, the specific activity of hTERT promoter and adenoviral transfection efficiency **A.** Representative histogram overlays of FACS analysis showed the isolated HUMSCs were positive for CD73, CD90 and CD105. **B.** Representative histogram overlays of FACS analysis showed the negative antigens of HUMSCs: CD34, CD19 and CD45. **C.** The representative microscope image showed differentiation of HUMSCs into the adipogenic lineages in vitro. Cells were stained with Oil-red O. **D.** The representative microscope image showed differentiation of HUMSCs into the osteogenic lineages in vitro. Cells were stained with Alizarin red. **(C, D)** scale bar = 200 μm. **E.** The schematic representation displayed the vectors involved in the study. **F.** The specific transcriptional activity of hTERT promoter in different cell lines was detected by a dual-luciferase reporter assay. **G.** Adenoviral transfection efficiencies at different MOI after 48 hours in three independent HUMSC samples were evaluated by FACS and expressed as mean ± SD. **H.** The representative images depicted the transfection efficiency of MSCs with 500 MOI Ad-Track. 48 hours after infection, HUMSCs carrying GFP were observed under fluorescent field (left panel) and bright field (right panel), scale bar = 100 μm.

The HUMSCs, obtained from the WJ of human umbilical cord with informed consents, had a typical spindle shape and resembled fibroblasts, consistent with the morphology reported by others [12, 27]. Although a specific antigen profile of HUMSCs has not been defined, for each isolation and culture, we verified by flow cytometry that the isolated cells were positive for the mesenchymal markers CD73, CD90 and CD105 (Figure 1A) and negative for typical hematopoietic antigens CD34, CD45 and CD19 (Figure 1B) as previously described [13]. Moreover, the HUMSCs were able to undergo adipogenesis (Figure 1C) and osteogenesis (Figure 1D) under specific differentiating conditions in vitro.

### The specific activity of hTERT promoter and the transfection efficiency of virus

We successfully cloned the specific hTERT promoter sequence (455bp), the IL-24 gene and the E1A gene, whose nucleotide sequences were verified by DNA sequencing. To examine the specific transcriptional activity of the hTERT promoter, we performed a dual-luciferase reporter assay using a pGL3-hTERTp reporter plasmid (Figure 1E). Luciferase activity in pGL3-hTERTp was expressed as a percentage of the positive control plasmid (pGL3-Control) driven by the SV40 enhancer/promoter. A pGL3-Basic plasmid without the enhancer/promoter sequence was used for negative control. HepG2, BJAB and K562 cells exhibited high transcriptional activity, whose values were 49.73±5.49%, 64.52±5.53%, and 134.59±16.25% of the positive control activity, respectively (Figure 1F). In contrast, neither normal human embryonic lung MRC-5 cells nor HUMSCs showed evident transcriptional activity, with values of 1.73±0.71% and 2.99±1.07% of the positive control activity, respectively. These findings suggested that the hTERT promoter we cloned had a specific transcriptional activity in cancer cells but not in normal cells.

Next, the lentiviral expression vectors pLentiR.E1A and the adenoviral expression vectors pAd-hTERTp-IL24 were successfully developed. The MOI of lentivirus of HUMSCs was set at 8 MOI as previously described [28]. We detected the adenoviral infection efficiency of HUMSCs using AdTrack at 48 h after infection. Flow cytometry revealed the efficiencies were 27.9±4.8%, 82.8±4.3% and 90.4±3.1% at MOI 100, 500 and 1000, respectively (Figure 1G). Based on these results, for the following experiments we chose 500 MOI, at which HUMSCs could be transfected efficiently without affecting their growth (Figure 1H).

### Replication-deficient adenoviruses amplified in HUMSC.LentiR.E1A and were eventually released from the cells

**Figure 2:**
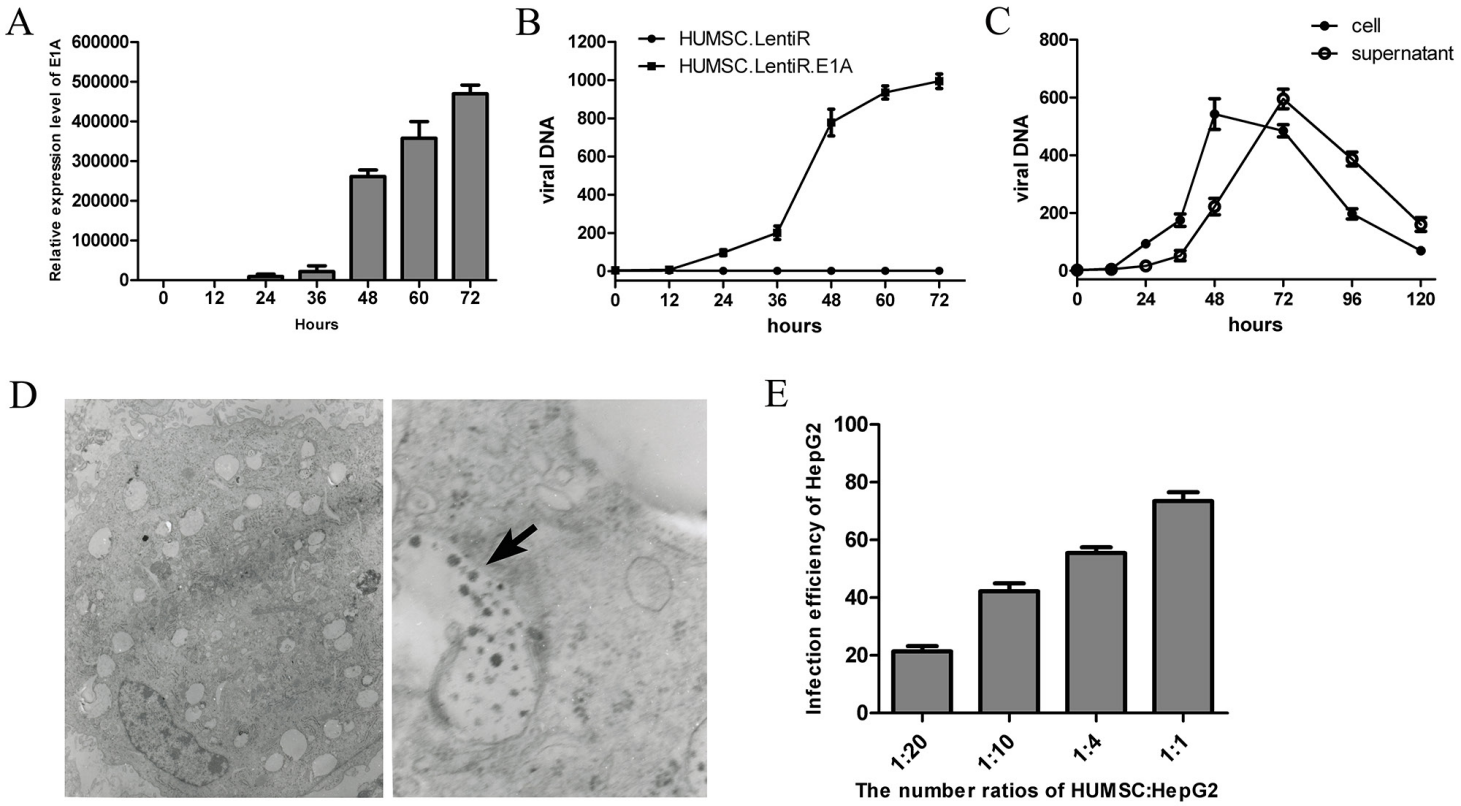
Replication-deficient adenoviruses amplified in HUMSC.LentiR.E1A and were eventually released from the cells **A.** The mRNA expression level of E1A at indicating time points after LentiR.E1A infection. **B.** The total concentration (intracellularly and in the supernatant) of viral DNA was measured at different time points in in three independent samples of HUMSC sequentially infected by Ad-Track and LentiR.E1A / LentiR. The start of LentiR.E1A infection was set as 0 h. **C.** The intracellular and supernatant concentration of viral DNA were detected at different time points respectively. **D.** Electron micrographs show viral particles in the HUMSC sequentially infected by Ad-Track and LentiR.E1A. Adenovirus particles were shown by the black arrow. Left panel: at low magnification; Right panel: at high magnification. **E.** The infection efficiency of HepG2 was detected by FACS 72 h after co-culture of virus-loaded (Ad-Track and LentiR.E1A) HUMSCs and HepG2 with different ratios in three independent samples.

To directly verify whether the replication-deficient adenovirus can be amplified in HUMSC.LentiR.E1A, we detected the concentrations of the hexon gene, a late adenoviral gene, intracellularly and in the supernatant of virus-loaded HUMSCs at the indicated time points after AdTrack and LentiR.E1A co-infection. Hexon gene expression was measured by quantitative PCR to determine the copy number of adenoviral DNA. The start of LentiR.E1A infection was set as 0 h. As the expression of E1A increased gradually (Figure 2A), the total concentration of adenoviral DNA (intracellularly and in the supernatant) in the HUMSC.LentiR.E1A group increased rapidly after 36 h, whereas the adenoviral DNA was not detected in the control group of HUMSC.LentiR (Figure 2B). In particular, the intracellular concentration of adenoviral DNA increased synchronously and sharply between 36 and 48 h after infection, and lasted until 72 h, while the supernatant concentration of viral DNA increased gradually and reached peak levels at 72 h in the HUMSC.LentiR.E1A group (Figure 2C). Further, electron microscopy verified the presence of intracellular viral particles in the HUMSCs 48 h after the infection (Figure 2D). These results indicated that viral DNA was synthesized and assembled into new viral particles in the HUMSCs co-infected by AdTrack and LentiR.E1A. Flow cytometry was used to detect the infection efficiency of HepG2 72 h after co-culture of virus-loaded HUMSCs and HepG2. The infection efficiencies were 21.3±3.3%, 42.3±4.7%, 55.5±3.5% and 72.4±3.9% at HUMSCs: HepG2 ratio of 1:20, 1:10, 1:4 and 1:1, respectively (Figure 2E). These observations indicated that the released viral particles have the ability to infect cells.

### Migration capacity of virus-loaded HUMSCs to hepatocarcinoma in vitro and in vivo

**Figure 3:**
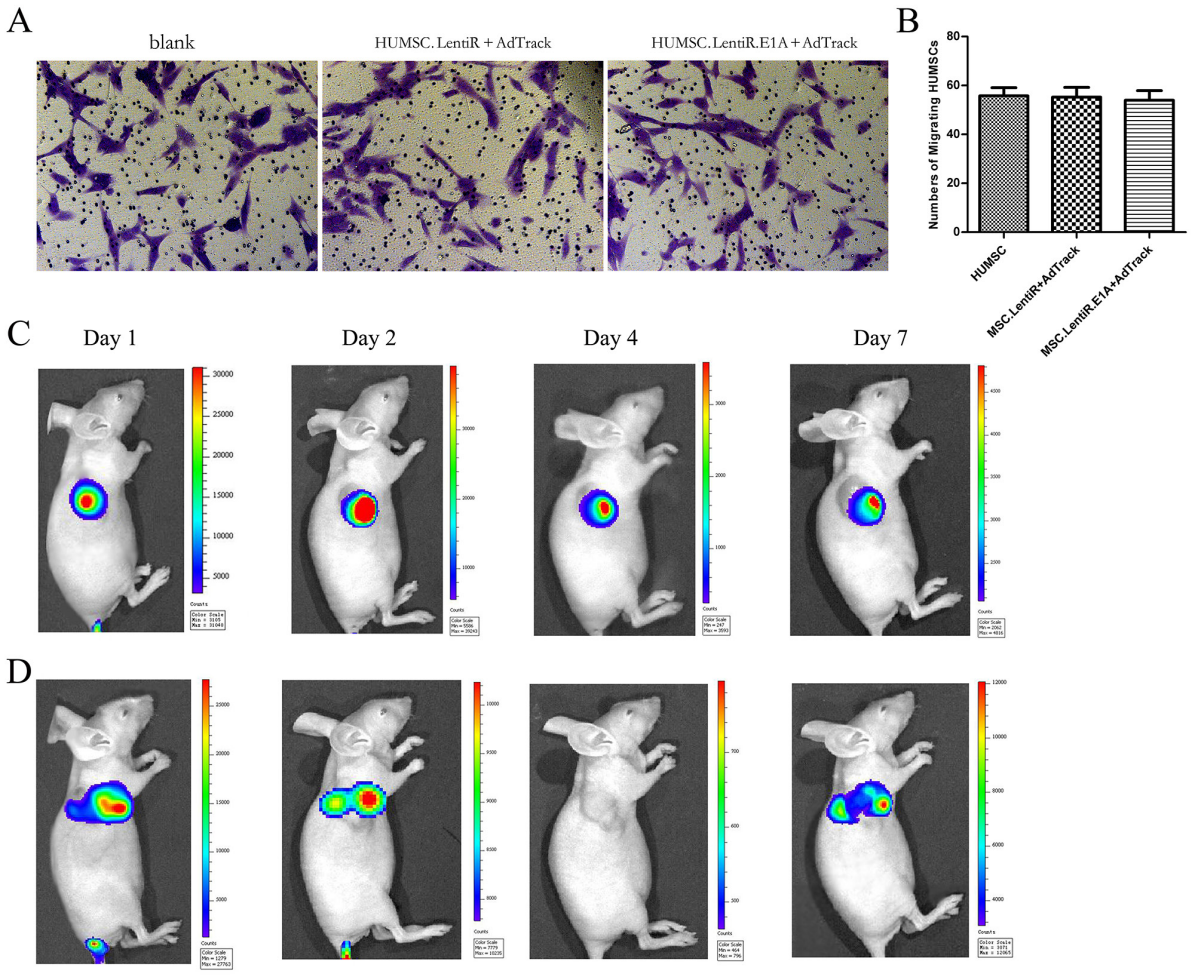
Migration capacity of virus-loaded HUMSCs to hepatocarcinoma in vitro and in vivo **A.** Representative photographs showed the migrated HUMSCs stained with crystal violet in vitro migration assays using Transwell plates. **B.** The numbers of migrated HUMSCs in three independent samples were expressed as mean ± SD. **C.** The homing capability of HUMSC.LentiR+Ad-Luc to tumor sites was monitored by bioluminescence imaging using Xenogen imaging system at indicated times after tail vein injection. **D.** The homing capability of virus-loaded HUMSC.LentiR.E1A+Ad-Luc to tumor sites was monitored in vivo.

HUMSCs and modified HUMSCs were previously shown to have a homing predisposition to tumor cells in vitro and to the tumor site in hepatocarcinoma models, and their migration capacity presents a concentration–dependent pattern [28]. However, in this study, the virus-loaded HUMSCs would be lysed after 36 h of LentiR.E1A infection because adenovirus replication and packaging was activated by the expression of E1A, as described in Figure 2B. To test the migration capacity of virus-loaded HUMSCs within 36 h after infection, in vitro migration assays using Transwell plates were performed. Unmodified HUMSCs were used as a positive control. We confirmed that virus-loaded HUMSCs migrated towards HepG2 cultures in a similar pattern as unmodified HUMSCs within 36 h after infection (Figure 3A, 3B). These results indicated that human hepatocellular carcinoma HepG2 cells were capable of stimulating the migration of HUMSCs and that the migration ability of HUMSCs was not affected by adenoviral and lentiviral co-infection.

Next, to investigate the homing capability of virus-loaded HUMSCs in vivo, we designed an adenoviral vector (pAd-Luc) harhoring a firefly luciferase reporter gene (Figure 1E). Representative Bioluminescent Imaging (BLI) analysis in vivo revealed that 1 day after injection of HUMSC.LentiR+Ad-Luc, intense firefly luciferase signal was detected at the tumors site. 2 days after the injection, the signal further increased, and it lasted until 7 days (Figure 3C). In the HUMSC.LentiR.E1A+Ad-Luc injection group, the intense imaging signal could also be detected at the tumor site 1 day after the injection, and disappeared after 4 days. The signal was discovered once again 7 days after the injection, indicating that the recombinant Ad-Luc was amplified and packaged in HUMSC.LentiR.E1A, and then released to infect the surrounding tumor cells (Figure 3D).

### Specific tumor suppressing effects of Ad-hTERTp-IL24 and low-dose 5-Fu could enhance growth inhibition of HepG2 cells

**Figure 4:**
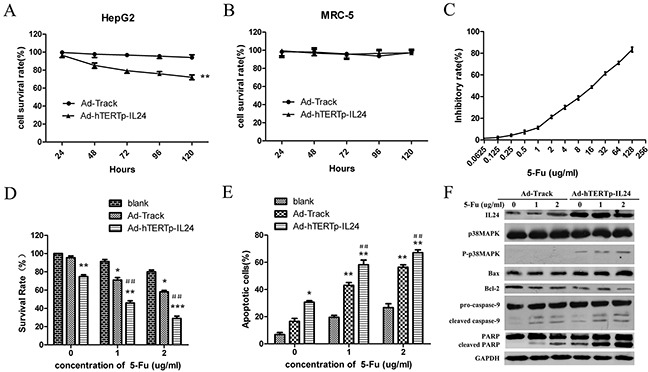
Specific tumor suppressing effects of Ad-hTERTp-IL24 and low dose of 5-Fu could enhance growth inhibition of HepG2 cells **A.** The cell viability of HepG2 cells was measured every 24 hours after infection with different adenoviruses at MOI 100 by CCK8 assays. The results were expressed as survival rate of untreated control.** *P*<0.01. **B.** The cell viability of MRC-5 cells was measured every 24 hours after infection with different adenoviruses at MOI 100 by CCK8 assays. **C.** The inhibitory rate curve of HepG2 cells treated with 5-Fu gradient concentrations was analyzed. **D.** The survival rates of HepG2 cells infected by adenoviruses at 100 MOI in the presence or absence of low doses of 5-Fu were tested by CCK8 assay and expressed as mean ± SD. *P<0.05, ** *P*<0.01 compared with 5-Fu treatment group; #*P*<0.05, ##*P*<0.01 compared with Ad-hTERTp-IL24 treatment group. **E.** Apoptosis ratios were detected when 5-Fu in combination with Ad-hTERTp-IL24 or AdTrack and expressed as mean ± SD. *P<0.05, ** *P*<0.01 compared with 5-Fu treatment group; #*P*<0.05, ##*P*<0.01 compared with Ad-hTERTp-IL24 treatment group. (F) Western blot showed proteins involved in apoptosis pathway when 5-Fu in combination with Ad-hTERTp-IL24 or AdTrack. All the data represent the averages of three independent experiments.

To evaluate the specific inhibitory effects of Ad-hTERTp-IL24 in tumor cells, CCK8 assays were performed at the indicated time points after infection. The results were expressed as survival rate of untreated control. The results showed that Ad-hTERTp-IL24 inhibited the growth of HepG2 cells (Figure 4A), but not of MRC-5 cells (Figure 4B), suggesting that the hTETR promoter, as well as IL-24, play a positive role only in tumor cells.

It has been reported that adenovirus in combination with 5-Fu or other chemotherapeutic reagents can efficiently eliminate cancer cells and tumors in vivo [28–30]. We further evaluated the inhibition efficacy of Ad-hTERTp-IL24 in combination with a low-dose of 5-Fu on HepG2 growth. Two concentrations of 5-Fu were used in combination with the adenovirus, 1 μg/ml and 2 μg/ml, corresponding to an inhibition rate of 11.30±1.27% and 21.25±1.34%, respectively (Figure 4C). The tumor cell killing ability was significantly increased when HepG2 were treated with Ad-hTERTp-IL24 in combination with 5-Fu, compared with Ad-hTERTp-IL24 or 5-Fu treatment alone (Figure 4D). Surprisingly, AdTrack combined with 5-Fu also caused more tumor cells death than 5-Fu treatment alone, as discussed in the next paragraph. To determine whether the adenovirus and 5-Fu interacted synergistically, the combination index (CI) of combination treatment was calculated. Strong synergistic cell killing was observed for 5-Fu in combination with Ad-hTERTp-IL24, and low synergism with AdTrack (Table 1). A similar synergistic effect was discovered in the apoptosis assays when 5-Fu was used in combination with Ad-hTERTp-IL24 or AdTrack (Figure 4E). Western blotting assays showed that IL-24 expression levels were greatly increased in the Ad-hTERTp-IL24 group, and promoted p38MAPK phosphorylation (Figure 4F), correlating cell killing with the activation of the p38MAPK pathway. Furthermore, Bax protein expression levels increased, while Bcl-2 protein expression decreased, and other apoptosis-related proteins such as PARP, caspase-3 and 9, were cleaved and activated. All of these changes were significantly more robust when Ad-hTERTp-IL24 and 5-Fu were combined (Figure 4F).

**Table 1:** Combination index (CI) of adenovirus combined with 5-Fu

5-FU (μg/ml)	Ad-Track (100 MOI)	Ad-hTERTp-IL24 (100 MOI)
1	0.85	0.75
2	0.73	0.64

### CAR and α_ν_β_3_ expression levels essential for virus internalization were increased in response to low doses of 5-Fu causing excessive adenoviral uptake

**Figure 5:**
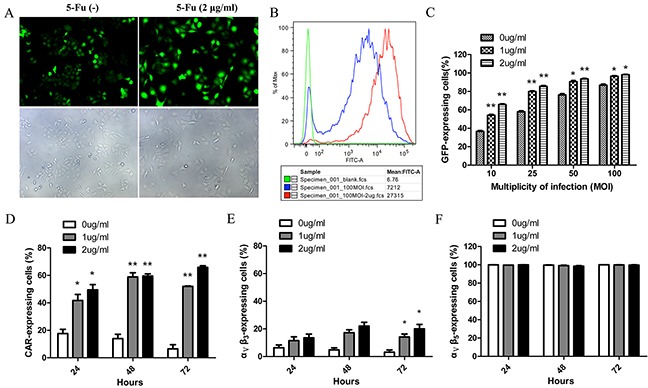
CAR and ανβ3 expression levels essential for virus internalization were increased in response to low doses of 5-Fu causing excessive adenoviral uptake **A.** Representative images showed the transfection of HepG2 with 100 MOI Ad-Track in the presence or absence of low-dose of 5-Fu (2μg/ml). 48 hours after infection, HepG2 cells were observed under fluorescent field (above panel) and bright field (below panel). **B.** Histogram overlays of flow cytometry revealed that the fluorescence intensity of each group in the Figure 5A. The red peak represented group of adenovirus in combined with 5-Fu; the blue peak represented group of adenovirus alone; the green peak represented group of negative control. **C.** The infection efficiencies of adenovirus for HepG2 at different MOIs were improved by low doses of 5-Fu. **D.** The expression level of CAR on the surface of HepG2 cells was detected every 24 hours after low doses of 5-Fu treatment. **E.** The expression level of α_ν_β_3_ on the surface of HepG2 cells was detected every 24 hours after low doses of 5-Fu treatment. **F.** The expression level of α_ν_β_5_ on the surface of HepG2 cells was detected every 24 hours after low doses of 5-Fu treatment. *P<0.05, ** *P*<0.01 compared with group of without 5-Fu treatment.

We observed that adenoviral infection efficiency was improved when combined with low doses of 5-Fu (Figure 5A). Flow cytometry analysis also showed that the fluorescence intensity of the combination treatment group was higher than the adenoviral treatment alone (Figure 5B). To test whether the synergistic effect was due to increased susceptibility to adenoviral infection in the presence of 5-Fu, HepG2 cells were infected with AdTrack at different MOIs in the presence or absence of low doses of 5-Fu. Dose-dependent increases in viral uptake from 10 to 100 MOI were observed, and significant increases in viral uptake were detected at all MOIs in the presence of low doses of 5-Fu (Figure 5C). Next, we investigated whether the expression levels of the viral attachment receptor CAR and the major internalization receptors α_ν_β_3_ and α_ν_β_5_integrins were increased in response to 5-Fu treatment, causing the higher viral uptake. In untreated HepG2 cells, the baseline CAR membrane levels reduced gradually with the increase of time in culture, and they were 17.60±4.38%, 13.95±4.45%, 6.53±3.31% at 24, 48 and 72 hours, respectively. While in the presence of 5-Fu, the cell surface expression levels of CAR significantly increased in HepG2 cells (Figure 5D). For integrin α_ν_β_3_, baseline expression was low (<10%), and it slightly increased after 5-Fu treatment (Figure 5E). For integrin α_ν_β_5_, baseline expression was as high as 100%, and 5-Fu treatment did not affect its expression levels (Figure 5F). All these observations might explain the excessive adenoviral uptake and the enhanced cytotoxicity when the adenovirus was combined with low doses of 5-Fu.

### Antitumor potential of Ad-hTERTp-IL24 loaded MSC.LentiR.E1A in combination with 5-Fu against HepG2 xenograft tumors

**Figure 6:**
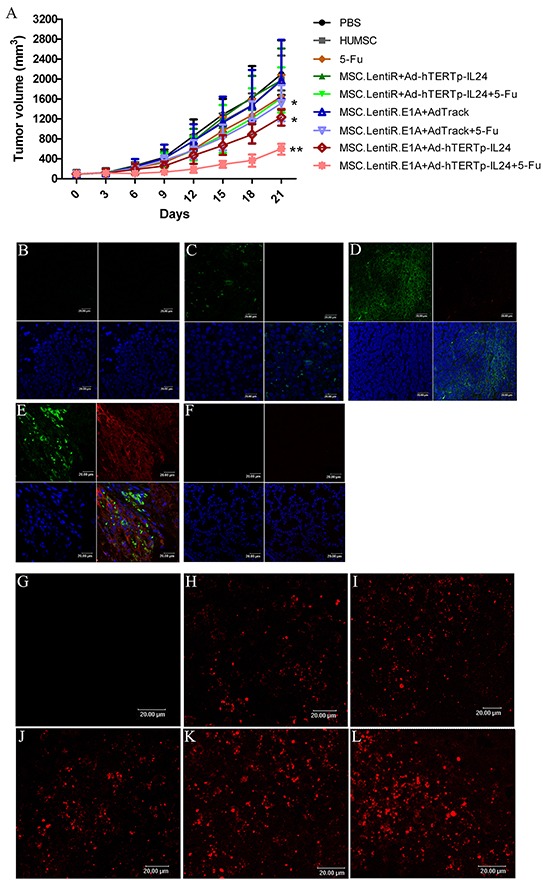
Tumor suppressing effects of Ad-hTERTp-IL24 loaded MSC.LentiR.E1A in combination with 5-Fu against HepG2 xenograft tumors **A.** The tumor volumes of different groups were measured every 3 days after treatment. Points indicate the mean values (*n*=5); bars indicate SD. *P<0.05, ** *P*<0.01 compared with PBS group. **B-F.** The target protein expression was detected in different groups by confocal microscopy. The red fluorescence represented IL-24 proteins; the green fluorescence represented the cells infected by adenovirus. The blue fluorescence showed the nuclei. (B) PBS; (C) MSC.LentiR+Ad-hTERTp-IL24; (D) MSC.LentiR.E1A+Ad-Track; (E) MSC.LentiR.E1A+Ad-hTERTp-IL24; (F) The lung tissue of the group of MSC.LentiR.E1A+Ad-hTERTp-IL24. **G-L.** TUNEL staining revealed apoptosis in the tumors of different treatment groups. Cy3-labeled TUNEL positive cells on the sections were detected by confocal microscopy. (G) PBS; (H) 5-Fu; (I) MSC.LentiR+Ad-hTERTp-IL24+5-Fu; (J) MSC.LentiR.E1A+Ad-Track+5-Fu; (K) MSC.LentiR.E1A+Ad-hTERTp-IL24; (L) MSC.LentiR.E1A+Ad-hTERTp-IL24+5-Fu.

We further investigated the antitumor potential of adenovirus-loaded MSC.LentiR.E1A in combination with 5-Fu in BALB/C nude mice transplanted with HepG2 human hepatocellular carcinoma cells. Compared with the mice in the control group (untreated), the mice treated with MSC.LentiR.E1A+Ad-hTERTp-IL24 with or without 5-Fu all exhibited evident tumor regression, especially in the MSC.LentiR.E1A+Ad-hTERTp-IL24 plus 5-Fu group, the tumor inhibition rate of which reached 71.21% 21 days after treatment (Figure 6A). Animals were sacrificed when tumors had grown to approximately 2000 mm^3^ in size. Tumors were dissected and fixed in 10% formalin for paraffin embedding.

Immunohistochemistry showed marked IL-24 expression (red) around the tumor cells (green) in the group treated with MSC.LentiR.E1A+Ad-hTERTp-IL24 (Figure 6E). In contrast, we could observe GFP-expressing cells negative for IL-24 expression among tumor tissues in the group of MSC.LentiR+Ad-hTERTp-IL24 (Figure 6C). In the MSC.LentiR.E1A+AdTrack group, GFP-expressing tumor cells lacking IL-24 expression were observed as expected (Figure 6D). Previous research has shown that a portion of transplanted HUMSCs are entrapped when injected intravenously [28, 31]. To investigate the fate of HUMSCs trapped by pulmonary capillaries in the lung, we also detected the expression of IL-24 and GFP in lung tissues treated with MSC.LentiR.E1A+Ad-hTERTp-IL24. Neither IL-24 nor GFP-expressing cells were detected in the lung (Figure 6F). The trapped HUMSCs had probably died rapidly due to the unsuitable environment, and they had released sporadic adenoviral particles in lung, indicating the tissue-specificity of this dual targeted system.

Further, TUNEL staining was performed to evaluate the apoptosis-inducing ability of this targeted system in vivo. Tumors treated with MSC.LentiR.E1A+Ad-hTERTp-IL24 with or without 5-Fu presented significantly apoptosis, which was limited to the tumor mass (Figure 6K,L). The apoptotic activity detected in other treated groups was much lower than in the MSC.LentiR.E1A+Ad-hTERTp-IL24 plus 5-Fu group (Figure 6G–6J), indicating that MSC.LentiR.E1A+Ad-hTERTp-IL24 and 5-Fu were synergistically induced tumor cell death in vivo.

## DISCUSSION

We successfully established a new targeted treatment system based on HUMSCs, which were engineered to deliver a replication-deficient adenoviral vector, and to enable its replication and assembling into new viruses to tumor sites in a mouse model of hepatocellular carcinoma. Our results showed that engineered HUMSCs have the ability to incorporate into tumors and release cancer-killing adenoviral particles. MSC.LentiR.E1A+ Ad-hTERTp-IL24 treatment exhibited significant anti-tumor effects on the transplanted hepatocarcinoma, which were mediated by Ad-hTERTp-IL24. Additionally, this tumor-suppressing effect was greatly strengthened when combined with low doses of 5-Fu, due to the increased expression of CAR and α_ν_β_3_ on tumor cells in response to low doses of 5-Fu. Previously, a similar targeted therapeutic strategy has been reported. Ando and his colleagues adapted a suicide system based on an inducible caspase-9 (iC9) protein that is activated using a specific chemical inducer of dimerization (CID) for adenoviral-based delivery to lung tumors through MSCs [32]. Their findings suggested that this approach could be used to kill NSCLC in vitro and in an orthotopic mouse xenograft model of lung cancer. Compared with this study, our results not only showed the effectively anti-tumor activity of this strategy but also provided more evidence of adenoviral replication in vitro and in vivo (Figures 2 and 3).

The tumor-migrating tropism of MSCs has been acknowledged in recent years. Due to its applications to tumor treatment, increasingly frequent investigations have been performed on the kinetic distribution of systemically administered MSCs in vivo and on the time required for MSCs to the tumor site [11, 33, 34]. Our laboratory previously demonstrated by luciferase bioluminescence in vivo that MSCs migrate and selectively accumulated at the tumor site at 24 h after intravenous injection [34]. Further, Xi Xia [33] investigated the distribution pattern of MSCs in a specific time period, ranging from 24 h to 72 h after injection, and found that the number of MSCs in the tumor increased over time. In this study, the therapeutic adenoviruses were delivered to tumor sites and amplified by HUMSCs sequentially infected by Ad-hTERTp-IL24 and LentiR.E1A. We found that the adenovirus could extensively amplify in HUMSCs 36 h after the transduction of LentiR.E1A in vitro. Hence, the lentiviral expression of E1A, which is necessary for adenoviral amplification, requires at least 36 h. The increased expression of E1A was accompanied by massive adenoviral amplification, and reached peak levels at 72 h. Furthermore, in vivo experiments demonstrated that it took no longer than 48 h for most of the virus-loaded HUMSCs to reach the tumor sites. These findings suggest that, taking advantage of the delay of lentiviral gene expression, a sufficient number of HUMSCs could migrate to tumor sites before being lysed.

Recently, MSCs have been demonstrated to deliver conditionally replicative adenovirus (CRAd) to various malignant tumors. These viruses are able to destroy tumor cells by replication and consequent oncolysis, which enables the newly produced virus to be released to the surrounding tumor tissues to prevent tumor growth [6, 11, 33, 35]. However, a considerable number of CRAd-loaded MSCs were also found in the lung, liver, and spleen, with the exception of the tumor site after systemic administration [33]. These ectopic CRAd-loaded MSCs would produce virus to injury normal tissues as well, due to their lack of absolute specificity, which limits the effective use of CRAd in clinical applications. In our study, we used E1A-engineered HUMSCs to deliver a replication-deficient adenovirus, which can replicate and assemble into new viruses to tumor sites. The replication-deficient adenovirus replicated only in HUMSCs but not tumor cells or other normal cells because of the complementary expression of E1A in HUMSCs.

Combining adenoviral constructs with chemothera-peutics has represented an appealing strategy to increase their potency [36]. Several studies have presented combinatory cytotoxic effects in esophageal carcinoma by Ad-delE1B55 in combination with 5-Fu [37], in pancreatic adenocarcinoma model by Ad-*dl*922-947 in combination with 5-Fu or gemcitabine [29], and in patients with recurrent head and neck cancer by intratumoral ONYX-015 in combination with cisplatin or 5-Fu [30]. The reasonable explanation for combinatory cytotoxic effects on tumors was that chemotherapeutic agents increased adenoviral infectivity dependent on CAR, α_ν_β_3_ and α_ν_β_5_ [29] or associated with morphological changes in lipid membranes [38]. In this study, we investigated the effects of 5-Fu, as it is frequently used for hepatocellular carcinoma treatment. Synergistic tumor cell inhibition was observed for 5-Fu in combination with Ad-hTERTp-IL24, as well as AdTrack in vitro and in vivo. Two explanations might account for the synergy: first, the expression level of CAR was increased in response to the 5-Fu treatment, causing the higher viral uptake; second, over-expressed IL-24 could enhance sensitivity of cancer cells to 5-Fu [39].

In conclusion, this work investigates a promising targeted therapeutic strategy using E1A-engineered HUMSCs to deliver and produce replication-deficient adenovirus against hepatocarcinoma to tumor sites. The therapeutic strategy provides a new, effective and safe administration route for replication-deficient adenovirus to resolve the problem of inefficient virus delivery to inaccessible and/or metastatic tumor sites. Meanwhile, it also solves the potential safety hazard of HUMSCs. However, this therapeutic system retains some problems that will need to be improved in the future. For example, the expression of E1A can be controlled by an inducible promoter, which would ensure more HUMSCs migrate to tumor sites before lysis, enhancing the tumor-suppressing effect. Additional studies are warranted to demonstrate the superiority of this therapeutic strategy in the tumor metastasis models, which would make it highly appealing for the treatment of tumor patients with metastatic diseases.

## MATERIALS AND METHODS

### Cell culture

The human hepatocellular carcinoma cell line HepG2 and the human embryonic lung fibroblast cell line MRC-5 were obtained from the Cell Resource Center, Peking Union Medical College (which is the headquarter of the National Infrastructure of Cell Line Resource, NSTI) on May 16th, 2012. Cells were tested for the absence of mycoplasma contamination by PCR and culture. Cell species was confirmed by PCR. The identity of the cell lines was authenticated with STR profiling (FBI, CODIS). All the results can be viewed on the website (http://cellresource.cn). Cells were maintained in DMEM supplemented with 10% FBS and α-MEM supplemented with 1×MEM Non-Essential Amino Acids and 10% FBS, respectively. The human embryonic renal cell line 293A (Institute of Hematology & Blood Diseases Hospital Chinese Academy of Medical Sciences & Peking Union Medical College, PUMC), and human embryonic kidney cell derived 293T cell line (kindly provided by Professor Cheng Tao, PUMC) were maintained in DMEM supplemented with 10% FBS. Cells were cultured in a cell incubator containing 5% CO_2_ at 37°C.

### HUMSCs preparation

HUMSCs were isolated from human umbilical cord Wharton's jelly (WJ) as previously described [27]. HUMSCs were seeded at a density of 8×10^3^ cells/cm^2^ in DF-12 supplemented with 2 mmol/l L-glutamine and 10% FBS. When cells reached 80~90% confluence, they were detached using a 0.125% trypsin/1 mM EDTA solution, and re-seeded using the same growth media for subsequent passages. Cells at passage number 3-5 were used for the following experiments.

### Recombinant adenovirus release from HUMSC.LentiR.E1A in vitro

HUMSCs were seeded in 6-well plates at a density of 1×10^5^ cells/well and incubated overnight at 37°C. On the next day, the HUMSCs were infected with Ad-hTERTp-IL24 at multiplicity of infection (MOI) 500 for 6 hours. Then, the culture medium was replaced with fresh medium containing lentiviral supernatants (LentiR or LentiR.E1A) at MOI 8 with 8μg/ml polybrene (Sigma, Santa Clara, CA). Twelve hours later, the medium was replaced. HUMSCs and supernatants were harvested after the indicated periods of lentiviral infection, and used for adenoviral DNA preparation using the High Pure Viral Nucleic Acid Extraction Kit (Roche, Basel, Switzerland). Quantitative real-time PCR was performed using ABI PRISM 7500 real time PCR system. SYBR green technology was used to detect a 286-bp-long amplicon (nucleotides 21049-21334) within the conserved region of the Ad5 hexon gene. The primers were designed as follows: 5′-GGTGGCCATTACCTTTGACTCTTC-3′ and 5′-CCACCTGTTGGTAGTCCTTGTATTTAGTATCATC-3′. PCR cycles were programmed according to the manufacturer's instructions for SYBR Premix Ex Taq reagent (Takara, Dalian, China). The standard curve for adenovirus quantification was generated by serial dilutions of pAdTrack plasmid. The experiments were repeated for three times.

### Migration assay of virus-loaded HUMSCs in vitro and in vivo

The migration of virus-loaded HUMSCs was determined using 8 μm pore membrane inserts with 6.5mm diameter (BD Falcon, New York, USA). 12 hours after co-infection, 1×10^5^ HUMSCs were plated in the top chamber in 400 μl of serum-free medium. The previous day, HepG2 cells were seeded at a density of 5×10^4^ cells/well in the lower chamber in fresh medium containing 2% FBS. After 20 h incubation at 37°C, cells that had not migrated from the upper side of the membrane were scraped off with a cotton swab, and membranes were stained with 0.1% crystal violet at 37°C for 45 min. Cells that had migrated to the lower side of the membrane were quantified. The number of cells was determined in five randomly selected high-power (×200) microscope fields. Experiments were performed in triplicate.

For the in vivo HUMSC migration assays, we developed an adenoviral vector containing a firefly luciferase reporter gene (pAd-Luc). Seven days after HepG2 cells inoculation into the mouse right armpits, when the solid tumors reached 100-200 mm^3^ in size, 1×10^6^ HUMSCs were infected by Ad-Luc at MOI 500, followed by LentiR.E1A infection for 8 hours, and injected into mice via the tail vein to detect cell migration. Bioluminescence imaging was performed using IVIS-Xenogen 100 system (Caliper Lifesciences, USA) at the indicated times, as previously described [28].

### Growth inhibition of hepatocellular carcinoma (HepG2) xenografts in vivo

All animal procedures were approved by the Committee on the use and care of animals, Chinese Academy of Medical Science. 5-6-weeks-old female BALB/c nude mice were inoculated subcutaneously with 5×10^6^ HepG2 cells into right armpits. 7 days after tumor inoculation, when solid tumors reached 100-200 mm^3^ in size, mice were randomized into 9 groups (7 mice for each group) as follows: (1) PBS control; (2) HUMSC; (3) 5-Fu; (4) MSC.LentiR+Ad-hTERTp-IL24; (5) MSC.LentiR+Ad-hTERTp-IL24 and 5-Fu; (6) MSC.LentiR.E1A+AdTrack; (7) MSC.LentiR.E1A+AdTrack and 5-Fu; (8) MSC.LentiR.E1A+Ad-hTERTp-IL24; (9) MSC.LentiR.E1A+Ad-hTERTp-IL24 and 5-Fu. The HUMSCs were co-infected as described above and injected intravenously with 1×10^6^ cells/mouse. 5-Fu was i.p. injected at a dose of 10 mg/kg for 5 continuous days starting 3 days after HUMSCs injection. Growing tumors were measured every three days using a vernier caliper in two perpendicular dimensions. The tumor volumes were calculated using the following formula: V=(L×W2)/2, where L represents the longest axis of the tumors (in mm) and W represents the axis perpendicular to L (in mm). Mice were sacrificed by cervical dislocation under anesthesia when the tumors reached 2000 mm^3^ in size, and tumor tissues were harvested and weighed.

### Immunohistochemical analysis for the expression of IL-24

Tumor tissues were fixed in 10% formalin and embedded in paraffin blocks. 5 μm sections were obtained for H&E staining and subsequent analysis. The expression of IL-24 in the tumors treated by MSC.LentiR.E1A+ Ad-hTERTp-IL24 was detected by immunohistochemistry. In brief, the paraffin sections were deparaffinized in xylene and rehydrated through a series of graded-ethanol and PBS solutions. Antigen retrieval was performed by heated in a hot bath. Sections were then treated with goat serum for 30 min at room temperature followed by incubation with rabbit anti-IL-24 monoclonal antibody (Abcam, Cambridge, UK) and mouse anti-GFP monoclonal antibody (Abbkine, CA) at 4°C overnight. On the following day, sections were incubated with secondary polyclonal donkey anti-rabbit DyLight 649-conjugated antibody and secondary polyclonal rabbit anti-mouse FITC-conjugated antibody for 30 min. 4, 6-diamidino-2-phenylindole (DAPI, Sigma, Santa Clara, CA) was used for nuclear staining. The stained sections were imaged by confocal microscopy (Leica TCS SP2, Germany).

### In situ analysis of apoptotic cells

Apoptotic cells in the tumors were detected by terminal deoxynucleotidyl transferase dUTP nucleotide nick end labeling (TUNEL). Briefly, a commercial one-step TUNEL apoptosis assay kit (Beyotime Institute of Biotechnology, Shanghai, China) was used according to the manufacturer's instructions. Cy3-labeled TUNEL positive cells on the sections were detected by confocal microscopy.

### Statistical analysis

Data were analyzed using an independent sample *t-*tests and were represented as the mean ± SD. *P*<0.05 was considered to be statistically significant, and *P*<0.01 was considered to be highly statistically significant.

## SUPPLEMENTARY MATERIALS AND METHODS



## Abbreviations

HCC: Hepatocellular carcinoma
TACE: Transcatheter arterial chemoembolization
Ad5: Adenovirus serotypes 5
MSCs: Mesenchymal stem cells
HUMSCs: Human umbilical cord-derived mesenchymal stem cells
Mda-7/IL24: Melanoma differentiation associated gene-7/interleukin-24
hTERT: Human telomerase reverse transcriptase
5-Fu: 5-fluorouracil
WJ: Wharton's jelly
CAR: Coxsackie adenovirus receptor
TUNEL: Terminal deoxynucleotidyl transferase dUTP nucleotide nick end labeling
MOI: Multiplicity of infection
CI: Combination index
CRAd: Conditionally replicating adenovirus.
