# Supplementary material for: E1A-engineered human umbilical cord mesenchymal stem cells as carriers and amplifiers for adenovirus suppress hepatocarcinoma in mice
Source: Oncotarget. 2016 Jun 17;7(32):51815–28. doi: 10.18632/oncotarget.10122 (PMC5239516; doi:10.18632/oncotarget.10122)
Supplement: Supplementary file 1 [file oncotarget-07-51815-s001.pdf]

E1A-engineered human umbilical cord mesenchymal stem cells as carriers and amplifiers for adenovirus suppress hepatocarcinoma in mice

SUPPLEMENTARY MATERIALS AND METHODS

HUMSCs characterization in vitro

For immunophenotypic analysis of the expanded cells, HUMSCs of passages 3-5 were analyzed by flow cytometry for the expression of stromal markers, including CD105, CD73 and CD90 (Biolegend, San Diego, CA) and the absence of hematopoietic markers, such as CD45, CD34 and CD19 (BD, New York). First, the HUMSCs were collected followed by washed twice with cold phosphate buffered saline (PBS). Then, 1×10^5 cells were incubated for 30 min in the dark with fluorescence-conjugated monoclonal antibodies: phycoerythrin (PE) labeled anti-CD73, anti-CD90, anti-CD34, anti-CD45, anti- CD19, allophycocyanin (APC) labeled anti-CD105 or mouse isotypes control, respectively. After being washed three times with cold PBS, the stained HUMSCs were then analyzed by flow cytometry (FACS LSRII, BD). The isotype antibodies were used as negative controls.

For adipogenic differentiation, the HUMSCs were maintained in IMDM medium containing 1 <M dexamethasone, 10 <g/ml insulin, 0.5 mM isobutyl methylxanthine, 60<M indomethacin and 10% FBS. Two weeks later, the cells were fixed and stained with Oil-red O. Osteoblast differentiation was performed by culturing HUMSCs in IMDM medium plus 0.1<M dexamethasone, 0.2 mM ascorbic acid-2-phosphate, 10 mM β-glycerophosphate and 10% FBS. All the reagent kits were bought from Sigma (Santa Clara, CA). Three weeks later, the cells were washed, fixed, and stained with Alizarin red.

Construction and production of recombinant adenovirus and lentivirus

The hTERT promoter fragment (-378 - +77) was amplified from K562 cell genomic DNA using the primer pairs as follows: 5′-CTCGAGTGGCCCCTCCCTCGGG-3′ and 5′-AAGCTTCGCGGGGGTGGCCGGGGC-3′, and then inserted into pGL3 basic vector to perform the dual-luciferase reporter assay for the transcriptional specificity of hTERT promoter as previously described [1]. The mda-7/IL24 gene coding region was cloned from lipopolysaccharide (LPS)-treated (1.5 <g/ml, 6 hours) human PBMCs from a healthy donor by reverse transcription-polymerase chain reaction (RT-PCR) using the following primers: 5′-GCTAGCGCCACCATGAATTTTCAACAG-3′ and 5′- GCGGCCGCTCAGAGCTTGTAGAATTTCTGC-3′. Overlap PCR was used to connect the fragment of IL24 with hTERT promoter, and then the resulted fusion product was inserted into the restriction enzyme sites XhoI and NotI of the adenovirus shuttle plasmid pAdTrack, which contained the enhanced green fluorescent protein (EGFP). Next, the Ad-hTERTp-IL24 was recombined, packaged and produced by methods previously described [2].

The E1A gene was amplified from 293A cells genomic DNA using the primer pairs as follows: 5′-GAATTCGCCACCATGAGACATATTATC-3′ and 5′- GGATCCTTATGGCCTGGGGCGTTTAC-3′, and then subcloned into the lentivector pCDH-DsRed (pLentiR) to generate the pLentiR.E1A. The lentiviral particles produced by 293T cells were conducted according to the System Biosciences (SBI, Canada) protocol. The titers of adenovirus and lentivirus were determined by TCID50. AdTrack and LentiR were used as negative control, respectively.

Cell viability assay

To prove the specificity of Ad-hTERTp-IL24 to tumor cells, HepG2 cells or MRC-5 were seeded in 96-well plate at the density of 5×10^3 cells per well. Next day, the cells were infected with AdTrack or Ad-hTERTp-IL24 at MOI 100. The cell viability was evaluated with CCK-8 (Dojindo, Kumamoto, Japan) every 24h after infection until 120 h later. The cells without infection were used as negative control.

For the study of combinatory antitumor effects of Ad-hTERTp-IL24 and chemotherapeutic agents 5-FU, HepG2 cells were seeded in 96-well plate, 5×10^3 cells per well. Next day, the cells were infected with AdTrack or Ad-hTERTp-IL24 at MOI 100, and treated with different concentrations (0, 1, 2 <g/mL) of 5-FU respectively. After 72h incubation at 37°C, 20 <l of CCK8 reagent was added to each well, cells were incubated in the dark at 37°C for another hour, and then optical density at 450 nm wavelength was measured by a Synergy H4 Hybrid Reader (BioTek, Vermont). The cells without treatment were used as negative control. Cell viability was calculated according the following

formula: cell viability (%) = OD_{450} of experimental/ OD_{450} of control ×100%.

Flow cytometry analysis

To measure the effect of 5-FU on transduction efficiency of adenovirus, HepG2 cells were infected by Ad-hTERTp-IL24 at 10, 25, 50, 100 MOI respectively, followed by treated with 5-FU at different concentrations (0, 1, 2 <g/ mL). The cells were harvested 48h after infection and the proportion of GFP-positive cells were determined by flow cytometry. For determination of the coxsackie adenovirus receptor (CAR) expression level, HepG2 cells were treated with 5-FU as mentioned above. At indicated time, cells were harvested, centrifuged, washed, and incubated for 1 h with rabbit polyclonal anti-CAR (Biosynthesis, Lewisville, Texas) at a 1:500 dilution. After washed three times with PBS, antibody-bound samples were incubated with secondary polyclonal donkey anti-rabbit DyLight 649-conjugated antibody (Biolegend, San Diego, CA) for 30min in the dark. Isotype controls were only incubated with secondary antibody. Samples were analyzed as above. The experiment was repeated for three times.

REFERENCES

1. Yan C, Yang M, Li Z, Li S, Hu X, Fan D, Zhang Y, Wang J and Xiong D. Suppression of orthotopically implanted hepatocarcinoma in mice by umbilical cord-derived mesenchymal stem cells with sTRAIL gene expression driven by AFP promoter. Biomaterials. 2014; 35:3035-3043.
2. Yuan XF, Peng HW, Ding YH, Yan CH, Zhang YJ, Yang M and Xiong DS. Gene therapy in B-NHL cell line using adenovirus-mediated transfer of secretable trimeric TRAIL gene expression driven by CD20 promoter. Experimental hematology. 2013; 41:221-230.
